# 4-[(*E*)-(5-*tert*-Butyl-2-hydroxy­phen­yl)diazen­yl]benzoic acid benzene hemisolvate

**DOI:** 10.1107/S1600536810003880

**Published:** 2010-02-06

**Authors:** Tushar S. Basu Baul, Anup Paul, Edward R. T. Tiekink

**Affiliations:** a Department of Chemistry, North-Eastern Hill University, NEHU Permanent Campus, Umshing, Shillong 793 022, India; b Department of Chemistry, University of Malaya, 50603 Kuala Lumpur, Malaysia

## Abstract

The title benzene hemisolvate, C_17_H_18_N_2_O_3_·0.5C_6_H_6_, features an essentially planar (the r.m.s. deviation of the non-H atoms, excluding methyl-C, is 0.071 Å) diazo mol­ecule with an *E* conformation about the N=N bond, and a half-mol­ecule of benzene disposed about a centre of inversion. The dihedral angle formed between the benzene rings of the diazo mol­ecule is 7.69 (12)°. In the crystal, centrosymmetrically related dimers associate *via* the eight-membered carboxylic acid dimer synthon, {⋯HOC(=O)}_2_, and these are connected into a supra­molecular chain along the *b* axis *via* C—H⋯O contacts.

## Related literature

For background to and motivation for the synthesis of the title compound, see: Basu Baul *et al.* (2010*a* ▶,*b* ▶,*c* ▶). For the structure of a related diazo compound, see: Basu Baul *et al.* (2008 ▶).
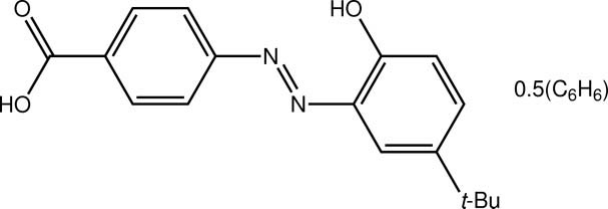


## Experimental

### 

#### Crystal data

C_17_H_18_N_2_O_3_·0.5C_6_H_6_
*M* *_r_* = 337.39
Triclinic, 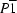

*a* = 6.0960 (2) Å
*b* = 7.3578 (3) Å
*c* = 20.6562 (7) Å
α = 81.326 (2)°
β = 88.992 (2)°
γ = 71.355 (2)°
*V* = 867.37 (5) Å^3^
*Z* = 2
Mo *K*α radiation
μ = 0.09 mm^−1^
*T* = 100 K
0.49 × 0.09 × 0.03 mm

#### Data collection

Bruker SMART APEXII diffractometer
Absorption correction: multi-scan (*SADABS*; Sheldrick, 1996 ▶) *T*_min_ = 0.859, *T*_max_ = 1
11445 measured reflections
3053 independent reflections
1888 reflections with *I* > 2σ(*I*)
*R*_int_ = 0.049

#### Refinement

*R*[*F* ^2^ > 2σ(*F* ^2^)] = 0.046
*wR*(*F* ^2^) = 0.157
*S* = 1.08
3053 reflections
232 parameters
H-atom parameters constrained
Δρ_max_ = 0.25 e Å^−3^
Δρ_min_ = −0.39 e Å^−3^

### 

Data collection: *APEX2* (Bruker, 2007 ▶); cell refinement: *SAINT* (Bruker, 2007 ▶); data reduction: *SAINT*; program(s) used to solve structure: *SHELXS86* (Sheldrick, 2008 ▶); program(s) used to refine structure: *SHELXL97* (Sheldrick, 2008 ▶); molecular graphics: *ORTEP-3* (Farrugia, 1997 ▶) and *DIAMOND* (Brandenburg, 2006 ▶); software used to prepare material for publication: *SHELXL97*.

## Supplementary Material

Crystal structure: contains datablocks global, I. DOI: 10.1107/S1600536810003880/hg2642sup1.cif

Structure factors: contains datablocks I. DOI: 10.1107/S1600536810003880/hg2642Isup2.hkl

Additional supplementary materials:  crystallographic information; 3D view; checkCIF report

## Figures and Tables

**Table 1:** Hydrogen-bond geometry (Å, °)

*D*—H⋯*A*	*D*—H	H⋯*A*	*D*⋯*A*	*D*—H⋯*A*
O3—H3o⋯N1	0.84	1.87	2.587 (3)	142
O2—H2o⋯O1^i^	0.84	1.79	2.614 (3)	167
C3—H3⋯O1^ii^	0.95	2.59	3.473 (3)	155
C6—H6⋯O3^iii^	0.95	2.48	3.201 (3)	133

Symmetry codes: (i) 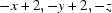; (ii) 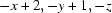; (iii) 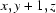.

**Fig. 1.**
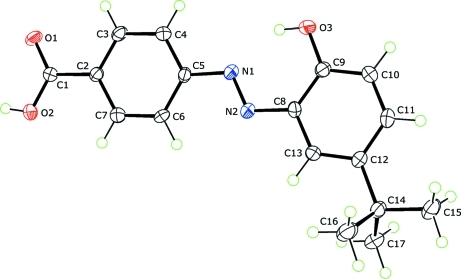
Molecular structure of (I) showing atom-labelling scheme and displacement ellipsoids at the 50% probability level.

**Fig. 2.**
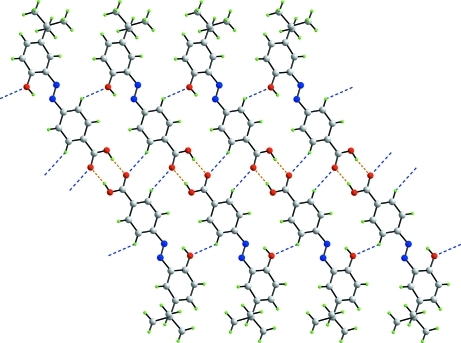
Supramolecular chain formation along the b axis in (I) mediated by O—H···O hydrogen bonds (orange dashed lines) and C—H···O contacts (blue dashed lines).

**Fig. 3.**
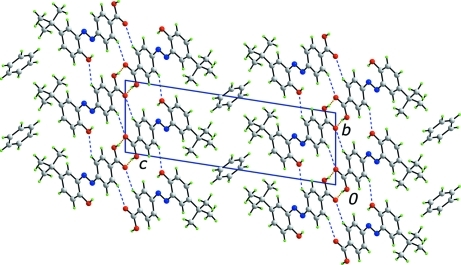
View in projection down the a axis of the crystal packing in (I), highlighting the stacking of supramolecular chains. The O—H···O hydrogen bonds are shown as orange dashed lines, and C—H···O contacts as blue dashed lines.

## supplementary crystallographic information

### Comment

4-Aminobenzoic acid reacts with 4-tert-butyl-phenol to yield the title compound which was prepared during an on-going study of the coordination chemistry of organotin carboxylates, their potential as cytotoxic agents and molecular modelling (Basu Baul *et al.*, 2010a,b,c). The asymmetric unit of (I) comprises a molecule of 4-[(*E*)-2-(5-tert-butyl-2-hydroxyphenyl)diazen-1-yl]benzoic acid and half a molecule of benzene, with the latter being disposed about a crystallographic centre of inversion. The conformation about the central N1═N2 bond [1.269 (3) Å] is *E*, Fig. 1. The diazo molecule is essentially planar with the exception of the *tert*-butyl group. The planarity is reflected in the sequence of O1–C1–C2–C3, C4–C5–N1–N2, and N1–N2–C8–C9 torsion angles of 6.4 (4), 172.6 (2), and 0.1 (4)°, respectively, which show only small twists in the molecule. The dihedral angle formed between the two benzene rings is 7.69 (12) °. The planar conformation is stabilised by an intramolecular O_hydroxyl_—H···N_azo_ hydrogen bond, Table 1. In terms of geometric parameters, the diazo compound in (I) resembles those reported for the 2-benzoic acid isomer (Basu Baul *et al.*, 2008).

The crystal packing features a familiar eight-membered carboxylic acid dimer synthon, Table 1. These are connected into a supramolecular chain via C–H···O contacts, Fig. 2 and Table 1. The C–H···O contacts involving the carbonyl-O2 atom lead to the formation of ten-membered {···HC_3_O}_2_ synthons, while those involving the hydroxyl-O3—H group are somewhat larger, i.e. 17-membered {···O_hydroxyl_C_2_N_2_C_3_H···O_carbonyl_···HO_carboxylic acid_C_4_H}_2_, and incorporate the intramolecular six-membered {···HOC_2_N_2_} synthons. The supramolecular chain has a flat topology and is aligned along the *b* axis. Chains stack in the crystal structure to form columns with the primary interactions between them being of the type π···π: ring centroid(C2—C7)···ring centroid(C8—C13)^i^ = 3.6637 (14) Å for *i*: 1+*x*, *y*, *z*. Interspersing these arrays are the solvent benzene molecules, Fig. 3.

### Experimental

The compound was prepared by reacting *p*-carboxybenzenediazonium chloride with 4-*tert*-butyl-phenol in alkaline solution under cold conditions (273–278 K) following the literature method (Basu Baul *et al.*, 2010a). Several crystallizations from methanol yielded red plates. M.pt.: 531–533 K. Anal. Calc. for C_17_H_18_N_2_O_3_: C, 68.44; H, 6.08; N, 9.39 %. Found. 68.39; H, 6.12; N, 9.40 %. IR (KBr): 1688 ν(OCO)_asym_. Single crystals suitable for an X-ray crystal-structure determination were obtained by slow evaporation of a benzene solution of the analytically pure compound and shown by crystallography to be the hemi-benzene solvate, (I).

### Refinement

All H-atoms were placed in calculated positions (O–H = 0.84 Å, and C–H = 0.95-0.98 Å) and were included in the refinement in the riding model approximation with *U*_iso_(H) set to 1.2-1.5*U*_eq_(carrier atom).

### Crystal data

**Table:**

C_17_H_18_N_2_O_3_·0.5C_6_H_6_	*Z* = 2
*M**_r_* = 337.39	*F*(000) = 358
Triclinic, *P*1	*D*_x_ = 1.292 Mg m^−^^3^
Hall symbol: -P 1	Mo *K*α radiation, λ = 0.71073 Å
*a* = 6.0960 (2) Å	Cell parameters from 2230 reflections
*b* = 7.3578 (3) Å	θ = 3.0–25.1°
*c* = 20.6562 (7) Å	µ = 0.09 mm^−^^1^
α = 81.326 (2)°	*T* = 100 K
β = 88.992 (2)°	Plate, orange
γ = 71.355 (2)°	0.49 × 0.09 × 0.03 mm
*V* = 867.37 (5) Å^3^	

### Data collection

**Table:**

Bruker SMART APEXII diffractometer	3053 independent reflections
Radiation source: sealed tube	1888 reflections with *I* > 2σ(*I*)
graphite	*R*_int_ = 0.049
φ and ω scans	θ_max_ = 25.0°, θ_min_ = 1.0°
Absorption correction: multi-scan (*SADABS*; Sheldrick, 1996)	*h* = −7→7
*T*_min_ = 0.859, *T*_max_ = 1	*k* = −8→8
11445 measured reflections	*l* = −24→24

### Refinement

**Table:**

Refinement on *F*^2^	Primary atom site location: structure-invariant direct methods
Least-squares matrix: full	Secondary atom site location: difference Fourier map
*R*[*F*^2^ > 2σ(*F*^2^)] = 0.046	Hydrogen site location: inferred from neighbouring sites
*wR*(*F*^2^) = 0.157	H-atom parameters constrained
*S* = 1.08	*w* = 1/[σ^2^(*F*_o_^2^) + (0.0815*P*)^2^ + 0.0458*P*] where *P* = (*F*_o_^2^ + 2*F*_c_^2^)/3
3053 reflections	(Δ/σ)_max_ = 0.001
232 parameters	Δρ_max_ = 0.25 e Å^−^^3^
0 restraints	Δρ_min_ = −0.39 e Å^−^^3^

### Special details

**Table:**

Geometry. All s.u.'s (except the s.u. in the dihedral angle between two l.s. planes) are estimated using the full covariance matrix. The cell s.u.'s are taken into account individually in the estimation of s.u.'s in distances, angles and torsion angles; correlations between s.u.'s in cell parameters are only used when they are defined by crystal symmetry. An approximate (isotropic) treatment of cell s.u.'s is used for estimating s.u.'s involving l.s. planes.
Refinement. Refinement of *F*^2^ against ALL reflections. The weighted *R*-factor wR and goodness of fit *S* are based on *F*^2^, conventional *R*-factors *R* are based on *F*, with *F* set to zero for negative *F*^2^. The threshold expression of *F*^2^ > 2σ(*F*^2^) is used only for calculating *R*-factors(gt) etc. and is not relevant to the choice of reflections for refinement. *R*-factors based on *F*^2^ are statistically about twice as large as those based on *F*, and *R*- factors based on ALL data will be even larger.

### Fractional atomic coordinates and isotropic or equivalent isotropic displacement parameters (Å^2^)

**Table:**

	*x*	*y*	*z*	*U*_iso_*/*U*_eq_	
O1	0.9406 (3)	0.7864 (2)	0.00097 (8)	0.0270 (5)	
O2	0.7757 (3)	1.0387 (2)	0.05359 (8)	0.0270 (5)	
H2O	0.8709	1.0827	0.0321	0.040*	
O3	0.0159 (3)	0.2070 (2)	0.17405 (9)	0.0295 (5)	
H3O	0.1019	0.2690	0.1569	0.044*	
N1	0.1696 (3)	0.4998 (3)	0.15671 (9)	0.0214 (5)	
N2	0.0282 (3)	0.5895 (3)	0.19601 (9)	0.0214 (5)	
C1	0.7939 (4)	0.8718 (4)	0.03965 (11)	0.0200 (6)	
C2	0.6296 (4)	0.7767 (3)	0.07027 (11)	0.0186 (6)	
C3	0.6505 (4)	0.5880 (4)	0.06190 (11)	0.0215 (6)	
H3	0.7704	0.5196	0.0362	0.026*	
C4	0.4971 (4)	0.5002 (4)	0.09100 (11)	0.0224 (6)	
H4	0.5120	0.3709	0.0856	0.027*	
C5	0.3208 (4)	0.6011 (4)	0.12827 (11)	0.0187 (6)	
C6	0.2955 (4)	0.7910 (4)	0.13581 (11)	0.0210 (6)	
H6	0.1722	0.8610	0.1602	0.025*	
C7	0.4513 (4)	0.8766 (4)	0.10745 (11)	0.0224 (6)	
H7	0.4371	1.0055	0.1133	0.027*	
C8	−0.1216 (4)	0.4932 (4)	0.22568 (11)	0.0193 (6)	
C9	−0.1262 (4)	0.3104 (4)	0.21544 (12)	0.0215 (6)	
C10	−0.2869 (4)	0.2363 (4)	0.24863 (12)	0.0254 (6)	
H10	−0.2942	0.1134	0.2419	0.030*	
C11	−0.4361 (4)	0.3394 (4)	0.29125 (12)	0.0245 (6)	
H11	−0.5437	0.2849	0.3134	0.029*	
C12	−0.4341 (4)	0.5213 (4)	0.30302 (11)	0.0201 (6)	
C13	−0.2753 (4)	0.5933 (4)	0.26916 (12)	0.0213 (6)	
H13	−0.2699	0.7169	0.2757	0.026*	
C14	−0.5974 (4)	0.6396 (4)	0.35012 (12)	0.0241 (6)	
C15	−0.7522 (5)	0.5308 (4)	0.38459 (13)	0.0357 (8)	
H15A	−0.8514	0.5080	0.3520	0.053*	
H15B	−0.8489	0.6083	0.4154	0.053*	
H15C	−0.6557	0.4060	0.4085	0.053*	
C16	−0.4527 (5)	0.6816 (4)	0.40241 (13)	0.0344 (7)	
H16A	−0.3585	0.5590	0.4281	0.052*	
H16B	−0.5559	0.7619	0.4314	0.052*	
H16C	−0.3511	0.7508	0.3811	0.052*	
C17	−0.7517 (5)	0.8319 (4)	0.31170 (13)	0.0307 (7)	
H17A	−0.6543	0.9044	0.2899	0.046*	
H17B	−0.8531	0.9088	0.3419	0.046*	
H17C	−0.8462	0.8052	0.2787	0.046*	
C18	0.8298 (5)	0.0370 (4)	0.45204 (13)	0.0313 (7)	
H18	0.7123	0.0620	0.4192	0.038*	
C19	0.9784 (5)	0.1460 (4)	0.44705 (13)	0.0305 (7)	
H19	0.9640	0.2458	0.4108	0.037*	
C20	1.1478 (5)	0.1084 (4)	0.49518 (13)	0.0334 (7)	
H20	1.2501	0.1831	0.4920	0.040*	

### Atomic displacement parameters (Å^2^)

**Table:**

	*U*^11^	*U*^22^	*U*^33^	*U*^12^	*U*^13^	*U*^23^
O1	0.0223 (10)	0.0252 (10)	0.0326 (10)	−0.0054 (8)	0.0128 (8)	−0.0077 (8)
O2	0.0259 (11)	0.0235 (11)	0.0361 (11)	−0.0136 (9)	0.0103 (9)	−0.0074 (9)
O3	0.0302 (11)	0.0227 (10)	0.0384 (11)	−0.0097 (9)	0.0153 (9)	−0.0123 (9)
N1	0.0173 (11)	0.0237 (12)	0.0241 (11)	−0.0065 (10)	0.0041 (9)	−0.0067 (10)
N2	0.0183 (11)	0.0236 (12)	0.0235 (11)	−0.0074 (10)	0.0046 (9)	−0.0062 (10)
C1	0.0195 (14)	0.0191 (14)	0.0220 (14)	−0.0065 (12)	0.0018 (11)	−0.0045 (11)
C2	0.0167 (14)	0.0205 (14)	0.0185 (13)	−0.0058 (11)	0.0021 (11)	−0.0038 (11)
C3	0.0192 (14)	0.0264 (16)	0.0190 (13)	−0.0059 (12)	0.0056 (11)	−0.0071 (11)
C4	0.0219 (14)	0.0221 (15)	0.0251 (14)	−0.0075 (12)	0.0046 (11)	−0.0087 (11)
C5	0.0154 (13)	0.0229 (15)	0.0198 (13)	−0.0082 (11)	0.0025 (11)	−0.0046 (11)
C6	0.0179 (14)	0.0221 (15)	0.0224 (13)	−0.0039 (12)	0.0072 (11)	−0.0080 (11)
C7	0.0228 (15)	0.0224 (15)	0.0232 (14)	−0.0079 (12)	0.0029 (12)	−0.0060 (12)
C8	0.0175 (13)	0.0217 (14)	0.0208 (13)	−0.0094 (12)	0.0004 (11)	−0.0032 (11)
C9	0.0194 (14)	0.0222 (15)	0.0226 (14)	−0.0054 (12)	0.0022 (11)	−0.0051 (11)
C10	0.0265 (15)	0.0208 (15)	0.0316 (15)	−0.0109 (13)	0.0044 (12)	−0.0059 (12)
C11	0.0212 (15)	0.0279 (16)	0.0260 (14)	−0.0118 (12)	0.0032 (12)	−0.0012 (12)
C12	0.0168 (13)	0.0231 (15)	0.0196 (13)	−0.0054 (11)	0.0013 (11)	−0.0027 (11)
C13	0.0201 (14)	0.0218 (14)	0.0245 (14)	−0.0079 (12)	0.0023 (11)	−0.0088 (11)
C14	0.0204 (14)	0.0304 (16)	0.0239 (14)	−0.0096 (12)	0.0056 (11)	−0.0085 (12)
C15	0.0292 (16)	0.0411 (18)	0.0372 (17)	−0.0118 (14)	0.0160 (14)	−0.0081 (14)
C16	0.0270 (16)	0.0456 (19)	0.0325 (16)	−0.0092 (14)	0.0073 (13)	−0.0177 (14)
C17	0.0257 (16)	0.0315 (17)	0.0335 (16)	−0.0048 (13)	0.0096 (13)	−0.0111 (13)
C18	0.0308 (17)	0.0334 (17)	0.0301 (16)	−0.0093 (14)	0.0007 (13)	−0.0084 (13)
C19	0.0381 (17)	0.0265 (16)	0.0270 (15)	−0.0099 (14)	0.0067 (13)	−0.0055 (12)
C20	0.0365 (18)	0.0378 (18)	0.0335 (16)	−0.0199 (15)	0.0076 (14)	−0.0114 (14)

### Geometric parameters (Å, °)

**Table:**

O1—C1	1.265 (3)	C11—C12	1.400 (4)
O2—C1	1.274 (3)	C11—H11	0.9500
O2—H2O	0.8400	C12—C13	1.379 (3)
O3—C9	1.345 (3)	C12—C14	1.537 (3)
O3—H3O	0.8400	C13—H13	0.9500
N1—N2	1.269 (3)	C14—C15	1.526 (3)
N1—C5	1.427 (3)	C14—C16	1.533 (4)
N2—C8	1.406 (3)	C14—C17	1.534 (4)
C1—C2	1.478 (3)	C15—H15A	0.9800
C2—C3	1.390 (3)	C15—H15B	0.9800
C2—C7	1.393 (3)	C15—H15C	0.9800
C3—C4	1.379 (3)	C16—H16A	0.9800
C3—H3	0.9500	C16—H16B	0.9800
C4—C5	1.390 (3)	C16—H16C	0.9800
C4—H4	0.9500	C17—H17A	0.9800
C5—C6	1.389 (3)	C17—H17B	0.9800
C6—C7	1.376 (3)	C17—H17C	0.9800
C6—H6	0.9500	C18—C20^i^	1.383 (4)
C7—H7	0.9500	C18—C19	1.383 (4)
C8—C13	1.398 (3)	C18—H18	0.9500
C8—C9	1.401 (3)	C19—C20	1.379 (4)
C9—C10	1.390 (3)	C19—H19	0.9500
C10—C11	1.382 (3)	C20—C18^i^	1.383 (4)
C10—H10	0.9500	C20—H20	0.9500
			
C1—O2—H2O	109.5	C13—C12—C14	120.3 (2)
C9—O3—H3O	109.5	C11—C12—C14	123.6 (2)
N2—N1—C5	114.0 (2)	C12—C13—C8	123.2 (2)
N1—N2—C8	115.6 (2)	C12—C13—H13	118.4
O1—C1—O2	123.8 (2)	C8—C13—H13	118.4
O1—C1—C2	119.2 (2)	C15—C14—C16	108.3 (2)
O2—C1—C2	117.0 (2)	C15—C14—C17	108.6 (2)
C3—C2—C7	119.5 (2)	C16—C14—C17	109.4 (2)
C3—C2—C1	120.4 (2)	C15—C14—C12	111.9 (2)
C7—C2—C1	120.1 (2)	C16—C14—C12	109.1 (2)
C4—C3—C2	120.0 (2)	C17—C14—C12	109.44 (19)
C4—C3—H3	120.0	C14—C15—H15A	109.5
C2—C3—H3	120.0	C14—C15—H15B	109.5
C3—C4—C5	119.9 (2)	H15A—C15—H15B	109.5
C3—C4—H4	120.0	C14—C15—H15C	109.5
C5—C4—H4	120.0	H15A—C15—H15C	109.5
C6—C5—C4	120.5 (2)	H15B—C15—H15C	109.5
C6—C5—N1	123.0 (2)	C14—C16—H16A	109.5
C4—C5—N1	116.4 (2)	C14—C16—H16B	109.5
C7—C6—C5	119.2 (2)	H16A—C16—H16B	109.5
C7—C6—H6	120.4	C14—C16—H16C	109.5
C5—C6—H6	120.4	H16A—C16—H16C	109.5
C6—C7—C2	120.8 (2)	H16B—C16—H16C	109.5
C6—C7—H7	119.6	C14—C17—H17A	109.5
C2—C7—H7	119.6	C14—C17—H17B	109.5
C13—C8—C9	119.4 (2)	H17A—C17—H17B	109.5
C13—C8—N2	114.9 (2)	C14—C17—H17C	109.5
C9—C8—N2	125.7 (2)	H17A—C17—H17C	109.5
O3—C9—C10	119.3 (2)	H17B—C17—H17C	109.5
O3—C9—C8	122.4 (2)	C20^i^—C18—C19	120.0 (3)
C10—C9—C8	118.3 (2)	C20^i^—C18—H18	120.0
C11—C10—C9	120.7 (2)	C19—C18—H18	120.0
C11—C10—H10	119.6	C20—C19—C18	119.3 (3)
C9—C10—H10	119.6	C20—C19—H19	120.3
C10—C11—C12	122.3 (2)	C18—C19—H19	120.3
C10—C11—H11	118.8	C19—C20—C18^i^	120.7 (3)
C12—C11—H11	118.8	C19—C20—H20	119.7
C13—C12—C11	116.1 (2)	C18^i^—C20—H20	119.7
			
C5—N1—N2—C8	−179.2 (2)	N2—C8—C9—O3	−0.9 (4)
O1—C1—C2—C3	6.4 (4)	C13—C8—C9—C10	0.8 (4)
O2—C1—C2—C3	−174.6 (2)	N2—C8—C9—C10	−179.7 (2)
O1—C1—C2—C7	−173.2 (2)	O3—C9—C10—C11	−179.7 (2)
O2—C1—C2—C7	5.8 (4)	C8—C9—C10—C11	−0.8 (4)
C7—C2—C3—C4	−0.8 (4)	C9—C10—C11—C12	0.2 (4)
C1—C2—C3—C4	179.6 (2)	C10—C11—C12—C13	0.5 (4)
C2—C3—C4—C5	0.5 (4)	C10—C11—C12—C14	−179.9 (2)
C3—C4—C5—C6	0.9 (4)	C11—C12—C13—C8	−0.5 (4)
C3—C4—C5—N1	−180.0 (2)	C14—C12—C13—C8	179.9 (2)
N2—N1—C5—C6	−8.3 (3)	C9—C8—C13—C12	−0.1 (4)
N2—N1—C5—C4	172.6 (2)	N2—C8—C13—C12	−179.7 (2)
C4—C5—C6—C7	−1.8 (4)	C13—C12—C14—C15	−177.7 (2)
N1—C5—C6—C7	179.1 (2)	C11—C12—C14—C15	2.7 (4)
C5—C6—C7—C2	1.5 (4)	C13—C12—C14—C16	−57.8 (3)
C3—C2—C7—C6	−0.2 (4)	C11—C12—C14—C16	122.6 (3)
C1—C2—C7—C6	179.4 (2)	C13—C12—C14—C17	61.9 (3)
N1—N2—C8—C13	179.6 (2)	C11—C12—C14—C17	−117.7 (3)
N1—N2—C8—C9	0.1 (4)	C20^i^—C18—C19—C20	−0.2 (4)
C13—C8—C9—O3	179.6 (2)	C18—C19—C20—C18^i^	0.2 (4)

Symmetry codes: (i) −*x*+2, −*y*, −*z*+1.

### Hydrogen-bond geometry (Å, °)

**Table:**

*D*—H···*A*	*D*—H	H···*A*	*D*···*A*	*D*—H···*A*
O3—H3o···N1	0.84	1.87	2.587 (3)	142
O2—H2o···O1^ii^	0.84	1.79	2.614 (3)	167
C3—H3···O1^iii^	0.95	2.59	3.473 (3)	155
C6—H6···O3^iv^	0.95	2.48	3.201 (3)	133

Symmetry codes:  (ii) −*x*+2, −*y*+2, −*z*; (iii) −*x*+2, −*y*+1, −*z*; (iv) *x*, *y*+1, *z*.
